# Analysis and Comparison of Nutrition Profiles of Canine Milk with Bovine and Caprine Milk

**DOI:** 10.3390/foods11030472

**Published:** 2022-02-05

**Authors:** Mengjie Zhang, Xiaomeng Sun, Jianjun Cheng, Mingruo Guo

**Affiliations:** 1 College of Food Science, Northeast Agricultural University, Harbin 150030, China; 18097867089@163.com (M.Z.); sunxm@neau.edu.cn (X.S.); jjcheng@neau.edu.cn (J.C.); 2 Department of Nutrition and Food Sciences, College of Agriculture and Life Sciences, University of Vermont, Burlington, VT 05405, USA

**Keywords:** canine milk, chemical composition, protein, fatty acids, mineral

## Abstract

Pet foods are gaining ground in China. Canine milk substitute formulations are based on their milk chemistry. This study aimed to analyze and compare the differences in proteins, fatty acids, minerals, and basic chemical composition between canine, bovine, and caprine milk. Canine milk contains higher contents of protein (6.62–17.34%), fat (8.92–14.31%), and ash (1.11–1.81%), and a lower content of lactose (1.56–3.92%) compared to bovine and caprine milk. The protein profiles of canine, bovine, and caprine milk were similar as confirmed by sodium dodecyl sulphate-polyacrylamide electrophoresis gel (SDS-PAGE). The quantification of proteins in canine, bovine, and caprine milk were significantly different when analyzed by inverse high-performance liquid chromatography. Canine milk showed higher contents of monounsaturated fatty acids (29.71–32.95% of total fatty acids) and polyunsaturated fatty acids (16.83–20.56% of total fatty acids), but a lower proportion of saturated fatty acids (47.57–53.18% of total fatty acids) than bovine and caprine milk. The essential fatty acids ARA and DHA were also found in canine milk in the ranges of 0.82–1.77% and 0.12–0.43% of total fatty acids, respectively. Canine milk had higher levels of Mg, K, Na, Fe, and Zn than those in bovine and caprine milk. The nutrient profile of canine milk was different from that of bovine and caprine milk. There were differences in nutritional compositions of the milk samples from four breeds, and Rottweiler milk had the highest nutritional content. The data of this study may provide useful information about the nutritional needs of puppies during their first months of life and the basic information for formulations of puppy milk substitutes.

## 1. Introduction

Increasing numbers of families raise dogs as partners and care for their health and growth. Breast milk is an essential source of nutrients (proteins, lipids, carbohydrates, vitamins, and minerals) for newborn puppies [1,2]. The bioactive composition of breast milk can regulate the development of the intestinal tract and immune system via its antimicrobial and anti-inflammatory effects [3,4]. When breastfeeding is unavailable, substitution with formula becomes an excellent alternative to meet the nutritional needs of puppies. It was reported that some publicly available milk powder substitutes may be deficient or excessive in certain nutrients, which can affect the development of puppies [5,6]. Therefore, it is necessary to perform systematic and comprehensive analyses of the nutrients and bioactive components in canine milk when developing formulations of puppy milk substitutes.

The nutrient profiles (protein, lipids, and carbohydrate content) of canine milk differ from those of other mammals [7]. Some active components in canine milk, including oligosaccharides (3′-sialyllactose, 6′-sialyllactose, and 2′-fucosyllactose), nucleotides (adenosine 5′-monophosphate, cytidine 5′-monophosphate, guanosine 5′-monophosphate, and uridine 5′-monophosphate), and immunoglobulin G were significantly different from other mammals [2,8,9]. However, the protein profile, fatty acid composition, and mineral content of canine milk are seldom reported.

Bovine and caprine milk are the main ingredients of the formulations of puppy milk alternatives [10]. Some clinical studies found that bovine colostrum reduced the recurrence rate of gastroenteritis in puppies and improved their fecal quality [11]. Bovine colostrum enhanced the immune response and the diversity and stability of intestinal microbes in dogs [12]. However, puppies have lower lactase activity in the small intestine after weaning, which leads to lactose intolerance [13]. Therefore, bovine and caprine milk cannot be used as a direct substitute for breast milk to meet the nutritional needs of puppies.

The objectives of this study were to analyze the chemical composition, including the protein profile, fatty acids, and minerals of canine milk from four breeds of dogs (Labrador, Caucasian Sheepdog, Golden Retriever, and Rottweiler), as well as bovine and caprine milk using sodium dodecyl sulphate-polyacrylamide gel electrophoresis (SDS-PAGE), reverse phase high-performance liquid chromatography (RP-HPLC), gas chromatography (GC), and inductively coupled plasma mass spectrometry (ICP-MS).

## 2. Materials and Methods

### 2.1. Materials

Standards of α-lactalbumin (purity > 85%), β-lactoglobulin (purity > 90%), and fatty acids (FAs) (Supelco 37-component FAME mix) were purchased from Sigma-Aldrich. The casein standard (purity > 95%) was obtained from the resource platform of the National Standard Material (Beijing, China). An internal standard (methyl salicylate) was purchased from Macklin (Shanghai, China). HPLC-grade acetonitrile, methanol, and n-hexane were purchased from the Fisher Scientific Company (Waltham, MA, USA). HPLC-grade chloroform and trifluoroacetic acid (TFA) were obtained from Aladdin. All other chemicals used were of analytical grade. The water used in this study was filtered through a Millipore Mill-Q water purification system (Millipore Corp., Milford, MA, USA).

### 2.2. Collection of Milk Samples

Bovine (Holstein) and caprine (Saanen) milk samples were purchased from a local farm in Harbin (China). Canine milk samples were collected from four breeds of healthy dogs (20–25 kg, 2–4 weeks of lactation) including Labrador (*n* = 4), Caucasian Sheepdog (*n* = 1), Golden Retriever (*n* = 1), and Rottweiler (*n* = 1). The milk obtained from different dogs was analyzed separately, except for the milk of the Labrador which was mixed for analysis. After manual collection, samples were immediately transferred to PET bottles, kept in an icebox at a temperature of 2–4 °C during transportation, and stored at −80 °C for further analysis.

### 2.3. Analysis of Basic Chemical Composition

All milk samples were thawed at 37 °C before chemical composition analysis. The protein content was determined using the micro-Kjeldahl method [14], and total proteins were obtained by multiplying the nitrogen percentage by a factor of 6.38. The fat content was measured according to IDF 105E (2008) [15]. Total solids (TS) were determined by the weight method after drying in a forced-draft oven at 105 °C until a steady weight was achieved according to IDF 021B (1987) [16]. Ash content was tested after the mineralization of milk at 550 °C for 4 h according to GB 5009.4 (2016) [17]. Lactose content was determined by the difference of total solids minus other solid components. All measurements were performed in triplicate.

### 2.4. SDS-PAGE Analysis

Milk samples for SDS-PAGE were prepared according to the method described by Wang et al. [18]. A concentrated gel (5%) and separated gel (12%) were prepared for the SDS-PAGE analysis. All the milk samples were diluted to 5 mg/mL using Milli-Q water (10 μL) and boiled for 5 min after mixing with 5 × SDS loading buffer CW0028S (Cwbiotechnology, Taizhou, China). Electrophoresis was conducted by a Mini-protean Tetra Electrophoresis System (Bio-Rad, Hercules, CA, USA). The gels ran at 85 V for 0.5 h in stacking gels and then 120 V for 1.5 h in separation gels. Protein staining was performed for 1 h using the Coomassie brilliant blue fast staining solution (Solarbio Co., Ltd, Beijing, China.). Ultrapure water was used for the decolorization treatment. A protein ladder ranging from 10 to 180 kDa (Biosharp Life Sciences, Hefei, China) was used as the molecular weight standard.

### 2.5. Analysis of Reverse Phase High Performance Liquid Chromatography (RP-HPLC)

Milk samples were skimmed by centrifugation at 10,000 rpm for 30 min at 4 °C, diluted with a mixed solution containing 8 M urea, 165 mM Tris, 44 mM sodium citrate, and 0.3% (*v*/*v*) β-mercaptoethanol [19]. Caprine and bovine milk were diluted 5-fold and canine milk was diluted 8-fold. After incubation at 37 °C for 1 h, samples were filtered through a 0.22-μm membrane for liquid chromatographic analysis. The identification and quantification of milk proteins were performed using a bovine milk protein standard containing α-lactalbumin (purity > 85%, Sigma), β-lactoglobulin (purity > 90%, Sigma), and casein (purity > 95%, National Standard Material Resource Platform, Beijing, China). The standards were prepared as 1, 2, 3, 4, and 5 mg/mL solutions with the above mixed solution.

An RP-HPLC analysis was performed according to the methods of Bobe et al. and Bekhit et al. [20,21] with some modifications using the Shimadzu DGU-20A3R liquid chromatography system (Shimadzu Co., Tokyo, Japan) equipped with an SPD-20A UV detector. Separations were performed on a reversed-phase analytical column C18 (Agilent TC-C18, Agilent Technologies) with 5-µm particle size (250 mm × 4.6 mm I.D.), and the column temperature was maintained at 40 °C. The flow phase A was deionized water containing 0.1% trifluoroacetic acid (TFA) and the flow phase B was acetonitrile solution containing 0.1% trifluoroacetic acid (TFA). The flow rate was 0.8 mL/min and the UV detection wavelength was set at 220 nm. The sample injection volume was 10 µL. The elution gradient conditions of the sample were set as follows: 0–5 min linear gradient from 33% B to 35% B; 5–9 min linear gradient from 35% B to 37% B; 9–18 min linear gradient from 37% B to 40% B; 18–22 min linear gradient from 40% B to 41% B; 22–27.5 isocratic elution 41%B; then 27.5–28 min linear gradient from 41% B to 43% B; 28–36 min linear gradient from 43% B to 45% B; 36–40 min linear gradient from 45% B to 33% B; followed by isocratic elution for 5 min at the initial conditions. The peak obtained from the standard solution was analyzed, and the standard curve between the peak area and the elution time was made. The major proteins in the milk samples were quantified and qualitatively analyzed by calculating the area of each peak and peak time of the sample, compared with the reverse HPLC elution map of the standard product.

### 2.6. Analysis of Fatty Acids (FAs)

Fatty acid composition was determined according to the method of Wang et al. [22], with some modifications. One hundred microliters of milk were mixed with 1 mL of chloroform/methanol (2:1, *v*/*v*) after recovery to room temperature. The mixed solution was ultrasonicated for 30 min and centrifuged at 10,000 rpm for 15 min at 4 °C. Two milliliters of sulfuric acid-methanol solution (1%) were added to the supernatant and methylated in an 80 °C bath for 30 min. The remaining solvent was blow dried with nitrogen. The dry sample was extracted by adding 1 mL of n-hexane and washed with 5 mL deionized water. The samples were prepared by mixing 475 μL supernatant with 25 μL internal standard (methyl salicylate) for GC analysis.

The fatty acid composition was determined by gas chromatography (Agilent 7890B) equipped with a flame ionization detector (FID) and Chromatography Workstation software. The fatty acid methyl esters (FAMEs) were separated on a capillary column SP-2560 (100 m × 0.25 mm ID × 0.25 µm). Hydrogen was used as a carrier gas with the flow of 1 mL/min, and the temperatures of injector and detector were 250 °C. All samples were injected with 1 μL, the split ratio was 100:1. The following GC conditions were used: the initial temperature was 140 °C, raised to 180 °C at a rate of 8 °C/min, raised to 210 °C at a rate of 8 °C/min, and then raised to 240 °C at a rate of 15 °C/min and maintained for 4.5 min, finally, raised to 250 °C at a rate of 5 °C/min and maintained for 10 min. The fatty acids in the samples were identified by comparing with the retention time of the standard methyl ester mixture. Fatty acid quantification was calculated by area normalization, and the amount of each fatty acid was expressed as a percentage of the total fatty acids.

### 2.7. Analysis of Minerals

The contents of calcium, magnesium, copper, iron, manganese, sodium, potassium, and zinc in all milk samples were analyzed by the standard method of GB5009.268 (2016) [23]. About 1 g of milk sample was treated with 5 mL of HNO_3_ in a microwave digestion tank, then heated on a microwave digestion apparatus at 120 °C for 5 min, 150 °C for 10 min, and 190 °C for 20 min. The solutions were cooled to room temperature and adjusted to a fixed volume to 25 mL with Milli-Q water. Mineral levels were determined by ICP-MS. Instrument operating parameters were radio frequency power 1500 W, coolant flow 11 L/min, auxiliary flow 0.40 L/min, and nebulizer flow 0.8 L/min. In addition, nickel/platinum cones were used as sampling cones with an integration time of 28 s, which were optimized for acid solutions.

### 2.8. Statistical Analysis

All analyses took three parallel measurements with data represented by the mean ± standard difference. A single-factor variance analysis was performed using SPSS 20 (IBM, New York, NY, USA) statistical software. Graphs were made using Origin 2021 (Origin Lab Corporation, Northampton, MA, USA).

## 3. Results and Discussion

### 3.1. Analysis of the Chemical Composition of Bovine, Caprine, and Canine Milk

The chemical composition of different milk samples is shown in Table 1. The composition of Rottweiler milk had a higher content than that reported by Park [24], which might be due to the differences in genetic properties, breeding, feeding conditions, the breed’s age, and calving number, diet, and lactation. There were significant differences in the nutritional composition of canine, bovine, and caprine milk (*p* < 0.05). Canine milk had twice the dry matter content than bovine and caprine milk due to its higher content of protein, fat, and ash. The average contents of the gross composition were 6.62–7.57% protein, 2.76–3.92% lactose, and 8.92–9.94% fat in Labrador, Caucasian Sheepdog, and Golden Retriever milk, which were in agreement with studies by Heinze et al. [25] and Oftedal et al. [26]. However, the ash concentration of canine milk was lower than that reported by Baines et al. [27]. The protein, fat, and ash content in Rottweiler milk was significantly (*p* < 0.05) higher than that of Labrador, Caucasian Sheepdog, and Golden Retriever milk. The intake of protein and fat has an important role in the growth and development of mammals. High protein levels could provide essential amino acids for the growth of newborns, and some special proteins can also improve immunity and promote the utilization of trace elements [28]. Fat, a major source of energy in breast milk, has high levels in canine milk, and is easily absorbed and utilized by the gut [29]. Lactose is a major carbohydrate in breast milk and provides energy. The lactose content of Golden Retriever, Labrador, and Rottweiler milk ranged from 1.56 to 2.86 g/100 mL, which was significantly lower than that of bovine and caprine milk (*p* < 0.05). The lactose content of Caucasian Sheepdog milk (3.92 g/100 mL) was similar to that of bovine (3.71 g/100 mL) and caprine milk (3.95 g/100 mL).

### 3.2. Protein Profiles of Bovine, Caprine, and Canine Milk by SDS-PAGE

**Figure 1:**
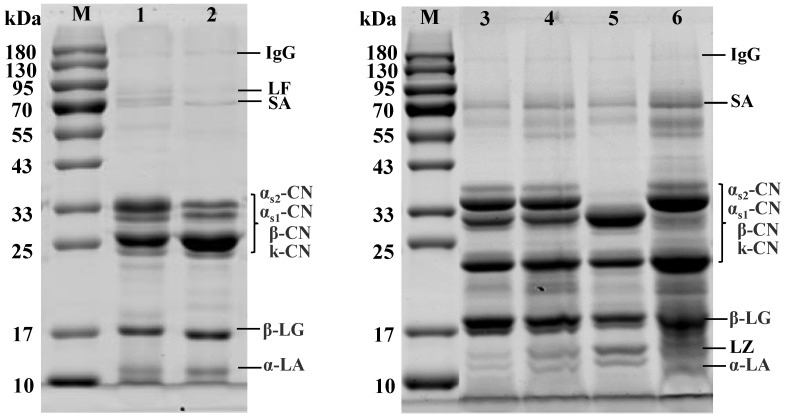
SDS-PAGE analysis of canine, bovine, and caprine milk. Lanes: M: Prestained protein molecular weight marker, (1) Holstein, (2) Saanen, (3) Golden Retriever, (4) Labrador, (5) Caucasian Sheepdog, (6) Rottweiler. LF: Lactoferrin, SA: Serum albumin, β-LG: β-lactoglobulin, LZ: Lysozyme, α-LA: α-lactalbumin, IgG: Immunoglobulin G, α_s1_-CN: α_s1_-casein, α_s2_-CN: α_s2_-casein, β-CN: β-casein, κ-CN: κ-casein.

The protein profiles of canine, bovine, and caprine milk were determined by SDS-PAGE (Figure 1). The profiles of bovine and caprine milk (Figure 1, lane 2 and 3) were equivalent to those reported by Ha et al. [30]. Milk proteins from four breeds of canines also showed good separation. Proteins observed in Figure 1 included α_s2_-casein (α_s2_-CN), α_s1_-casein (α_s1_-CN), β-casein (β-CN), κ-casein (κ-CN), α-lactalbumin (α-LA), β-lactoglobulin (β-LG), lysozyme, and serum albumin. The molecular weight of casein ranged from 19 to 35 kDa.

As shown in Figure 1, the distribution of whey proteins in canine milk was similar to that in bovine and caprine milk. A significant lysozyme band was observed in canine whey, and Halliday et al. [31] also found that the lysozyme content in canine milk was 2.57 ± 0.27 mg/mL, and there was no significant difference in the amino acid sequence between canine milk and human milk. The bands of lactoferrin in canine milks were weaker than those in bovine and caprine milk. β-LG was the most pronounced protein in canine whey and seemed to have a higher molecular weight than that of bovine and caprine β-LG. The α-LA band was relatively weak. The α_s2_-CN band was stronger than that of α_s1_-CN. Compared with bovine milk, the α_s1_-CN band in canine milk was weaker. The band width of β-CN was smaller than that of bovine and caprine milk. The distribution of the milk proteins, β-CN, κ-CN, and α-casein (α-CN) were markedly different in Caucasian Sheepdog milk compared with the other three breeds.

### 3.3. Identification of Proteins in Canine, Bovine, and Caprine Milk by RP-HPLC

**Figure 2:**
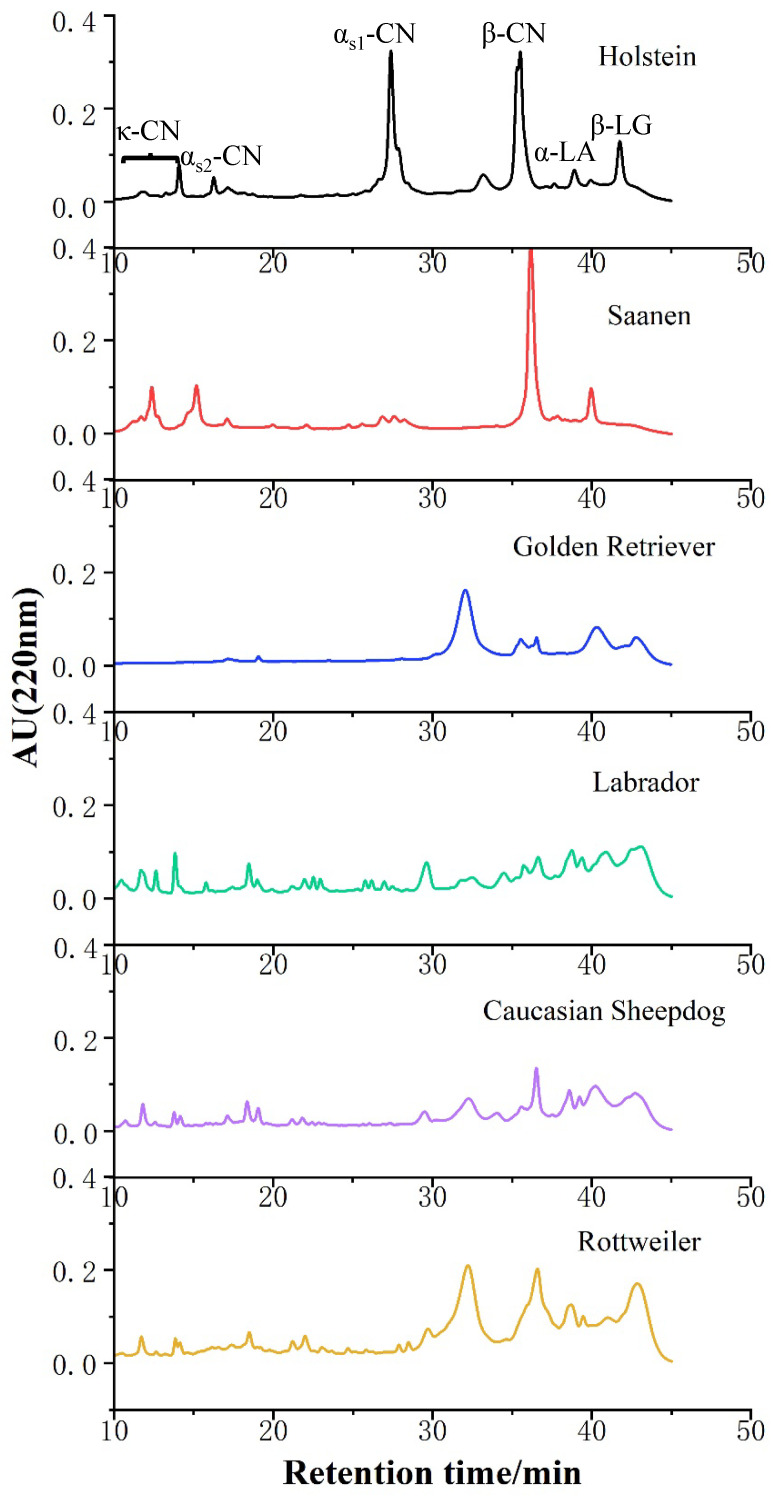
Reverse phase high-performance liquid chromatography chromatograms of protein fractions in canine, bovine, and caprine milk. α_s1_-CN: α_s1_-casein, α_s2_-CN: α_s2_-casein, β-CN: β-casein, κ-CN: κ-casein, β-LG: β-lactoglobulin, α-LA: α-lactalbumin.

The protein profiles of canine, bovine, and caprine skim milk were determined by RP-HPLC (Figure 2). Canine casein, α-LA, and β-LG showed different separation compared with bovine and caprine milk, which might be due to the hydrophobic interactions of the proteins as well as amino acid composition. The elution profiles of bovine and caprine milk proteins in Figure 2 were consistent with those reported by Bonfatti et al. and Moatsou et al. [21,32].

The relative proportions of milk proteins were associated with species and lactation stage [33], which were responsible for the differences in the chromatograms. As shown in Figure 2, the milk protein chromatograms of different canine milks were similar, but the κ-CN and α_s2_-CN levels in Golden Retriever milk were not well separated. Because the standards used in this study were bovine casein, the retention time of α-CN in canine milk shifted compared to bovine and caprine milk. The peak-time ranges of α_s2_-CN and α_s1_-CN were 18.12–18.73 min and 31–33.68 min according to the results of the chromatogram and SDS-PAGE. Differences in the elution time of proteins from different species were attributed to different amino acid compositions and modification of the proteins [34].

The quantification data calculated based on the peak areas of the chromatographic profiles were presented in Table 2. The contents of α_s1_-CN, α_s2_-CN, β-CN, κ-CN, β-LG, α-LA were the significant differences in canine, bovine, and caprine milk (*p* < 0.05).

The content of κ-CN in canine milk could not be estimated due to a shift in elution profile, and four variants of κ-CN have been identified in bovine milk. α_s1_-CN is the major casein in canine casein, followed by β-CN. There was a significantly higher content of β-LG and α-LA in canine whey protein than in bovine and caprine milk. α_s1_-CN and β-LG in bovine milk are major factors in infant allergy, whereas the surface hydrophobic amino acids of β-LG in caprine milk are easier to decompose, explaining why caprine milk has lower sensitization and higher digestibility than bovine milk [35,36,37]. However, the role of these protein components in puppies remains to be investigated and analyzed.

### 3.4. The Fatty Acid Profile Composition of Canine, Bovine, and Caprine Milk 

The compositions and contents of FAs in different milk samples are shown in Table 3. The fatty acid profiles were significantly different between the milk samples (*p* < 0.05). In ruminant milk, approximately half of the long-chain FAs (≥ C18) were derived from the diet. The majority of smaller C4:0-C14:0 FAs originate from the *de novo* FA synthesis in the mammary gland, but C16 can be synthesized from the diet and the mammary glands [38]. The unsaturated fatty acid content, especially C6-C12 FAs in canine milk, was lower than that in bovine and caprine milk. Palmitic acid (C16:0) was the most abundant saturated fatty acid accounting for 38.5%, 36.71%, and 30.03–33.96% in bovine, caprine, and canine milk, respectively. There was no significant difference in palmitic acid content between the different breeds of dog (*p* > 0.05), except for Rottweiler. Among saturated fatty acids (SFAs), the content of short chain fatty acids (C4-C8) and medium-long chain fatty acids (C10-C20) in canine milk were lower than those of bovine and caprine milk. It was reported that short chain fatty acids play a major role in infant health by regulating lipid metabolism, and intestinal flora by adjusting the intestinal pH [39,40].

The proportions of monounsaturated fatty acids (MUFAs) in bovine, caprine, and canine milk were 26.61%, 24.1%, and 29.71–32.95%, respectively, which were significantly different (*p* < 0.05). Oleic acid (C18:1) was the predominant MUFA in milk samples, which was an important source of energy for breast-fed infant [41,42]. The same result was found in canine milk (27.33–29.28%). Polyunsaturated fatty acids (PUFAs), especially n-3 and n-6 PUFAs, in canine milk were significantly higher than in bovine and caprine milk (*p* < 0.05). Regarding n-6 PUFAs, linoleic (C18:2, LA) was dominant in breast milk, followed by arachidonic acid (C20:4, ARA). Koletzko et al. [43] reported that docosahexaenoic acid (C22:6n-3, DHA) and ARA were the main components of membrane phospholipids, and play important roles in infant retinal, brain, platelet, and immune system development. Bauer et al. [44] found that puppies fed with a supplement of long chain n-3 fatty acids (EPA and DHA) had better vision than those fed on a non-supplemented control diet. Zicker et al. [45] reported that puppies fed with DHA after weaning had better learning, cognitive ability, and memory, as well as improved immunity and retinal function.

### 3.5. Mineral Contents in Canine, Bovine, and Caprine Milk

Minerals regulate the biochemical cells of the body, and an excess or deficiency of minerals is harmful to the health and production of livestock [46]. The mineral content of different milk samples is shown in Table 4. The contents of Mg, Na, Fe, Zn, Cu, Mn, and Se in canine milk were higher than in bovine and caprine milk, but the content of K was significantly lower (*p* < 0.05). Haenlein et al. [47] reported that caprine milk had similar contents of Na, Fe, Zn, and Mn to bovine milk, but a higher content of Ca, K, Mg, P, and Cl.

There were significant differences in mineral composition between different canine milk samples (*p* < 0.05). K, Fe, Zn, Cu, and Se content in Rottweiler milk was significantly higher than in Golden Retriever, Labrador, and Caucasian Sheepdog milk. According to Table 4, K (76.9–113.3 mg/100 mL) content in canine milk was the most abundant mineral, followed by Na (80.7–110.2 mg/100 mL). The contents of Zn (0.85–1.34 mg/100 mL) and Mg (9.85–13 mg/100 mL) were higher, the content of Cu (0.046–0.119 mg/100 mL) was lower, and the content of Fe (0.405–0.749 mg/100 mL) was similar in this study compared with the results reported by Anderson et al. [48]. Differences in mineral content in canine milk may be related to breed, feed, lactation stage, or different analysis methods.

## 4. Conclusions

There were differences in the nutrient profiles of canine milk compared with bovine and caprine milk. The nutrient contents in four canine milk samples were different, and the milk obtained from the Rottweiler had the highest density of nutrients. Canine milk had higher protein, fat, and ash content, and lower lactose content than bovine and caprine milk. The protein profile of canine milk was similar to bovine and caprine milk, but the protein content was considerably different. Canine milk had higher levels of unsaturated fatty acids including the essential fatty acids LA, ARA, DHA, and lower proportions of SFAs than bovine and caprine milk. Canine milk contained more minerals than bovine and caprine milk. This study may provide useful information about the composition of canine milk and may provide insights for the development of canine milk substitute formulations.

## Figures and Tables

**Table 1:** The chemical composition of canine, bovine, and caprine milk (g/100 mL).

Nutrient	Holstein	Saanen	Golden Retriever	Labrador	Caucasian Sheepdog	Rottweiler
Moisture	88.64 ± 0.11 ^a^	87.62 ± 0.1 ^a^	79.41 ± 0.16 ^b^	78.45 ± 0.65 ^b^	78.79 ± 0.08 ^b^	64.97 ± 0.47 ^c^
Dry matter	11.36 ± 0.11 ^a^	12.38 ± 0.1 ^a^	20.59 ± 0.16 ^b^	22.23 ± 1.73 ^c^	21.21 ± 0.08 ^bc^	35.03 ± 0.47 ^d^
Crude protein	3.12 ± 0.14 ^a^	3.09 ± 0.05 ^a^	7.57 ± 0.5 ^b^	8.34 ± 0.06 ^c^	6.62 ± 0.06 ^d^	17.34 ± 0.28 ^e^
Fat	3.71 ± 0.24 ^a^	4.45 ± 0.31 ^b^	8.92 ± 0.17 ^c^	9.94 ± 0.52 ^d^	9.56 ± 0.28 ^d^	14.31 ± 0.29 ^e^
Ash	0.82 ± 0.01 ^a^	0.88 ± 0.02 ^a^	1.23 ± 0.04 ^c^	1.19 ± 0.08 ^bc^	1.11 ± 0.04 ^b^	1.81 ± 0.05 ^d^
Lactose	3.71 ± 0.25 ^a^	3.95 ± 0.38 ^a^	2.86 ± 0.64 ^ab^	2.76 ± 1.53 ^ab^	3.92 ± 0.33 ^a^	1.56 ± 0.33 ^b^

Values within each row with different superscripts were significantly different (*p* < 0.05). Data are presented as the mean ± standard deviation, *n* = 3.

**Table 2:** Concentration of milk proteins of canine, bovine, and caprine milk (mg/mL).

	Holstein	Saanen	Golden Retriever	Labrador	Caucasian Sheepdog	Rottweiler
α_s1_-CN	9.45 ± 0.37 ^b^	3.16 ± 0.44 ^a^	9.67 ± 0.60 ^b^	4.27 ± 0.24 ^a^	7.76 ± 1.04 ^b^	30.98 ± 3.95 ^c^
α_s2_-CN	1.86 ± 0.04 ^a^	7.26 ± 0.28 ^b^	ND	4.83 ± 0.78 ^c^	4.30 ± 0.43 ^c^	6.73 ± 0.63 ^b^
β-CN	6.59 ± 0.31 ^a^	9.19 ± 0.66 ^b^	2.27 ± 0.83 ^c^	5.19 ± 0.46 ^d^	6.50 ± 0.49 ^a^	17.26 ± 0.17 ^e^
κ-CN	3.20 ± 0.02 ^a^	5.74 ± 0.16 ^b^	ND	ND	ND	ND
β-LG	3.35 ± 0.82 ^a^	2.79 ± 0.76 ^a^	7.84 ± 1.25 ^b^	12.56 ± 3.11 ^d^	11.5 ± 0.73 ^cd^	8.69 ± 0.88 ^bc^
α-LA	0.78 ± 0.18 ^a^	0.67 ± 0.07 ^a^	0.92 ± 0.10 ^a^	4.08 ± 0.18 ^b^	3.59 ± 0.35 ^c^	4.99 ± 0.49 ^d^

Values within each row with different superscripts are significantly different (*p* < 0.05). Data are presented as the mean ± standard deviation, *n* = 3. α_s1_-CN: α_s1_-casein, α_s2_-CN: α_s2_-casein, β-CN: β-casein, κ-CN: κ-casein, β-LG: β-lactoglobulin, α-LA: α-lactalbumin. ND = Not detected.

**Table 3:** Fatty acid profiles of canine, bovine, and caprine milk (g/100 g total fatty acid).

	Holstein	Saanen	Golden Retriever	Labrador	Caucasian Sheepdog	Rottweiler
C4:0	0.48 ± 0.11 ^a^	0.64 ± 0.19 ^a^	0.25 ± 0.08 ^b^	0.24 ± 0.04 ^b^	0.26 ± 0.11 ^b^	0.15 ± 0.06 ^b^
C6:0	0.94 ± 0.18 ^ab^	2.06 ± 0.31 ^c^	0.76 ± 0.04 ^ab^	0.82 ± 0.1 ^ab^	0.62 ± 0.06 ^a^	1.01 ± 0.25 ^b^
C10:0	0.78 ± 0.04 ^b^	2.43 ± 0.48 ^a^	0.30 ± 0.1 ^c^	0.17 ± 0.03 ^c^	0.43 ± 0.03 ^bc^	0.19 ± 0.03 ^c^
C12:0	2.29 ± 0.08 ^a^	2.94 ± 0.21 ^b^	0.31 ± 0.02 ^d^	0.09 ± 0.01 ^e^	0.69 ± 0.03 ^c^	0.10 ± 0.02 ^e^
C14:0	13.36 ± 0.6 ^a^	13.93 ± 0.54 ^a^	3.25 ± 0.08 ^c^	3.23 ± 0.7 ^c^	5.28 ± 0.14 ^b^	1.63 ± 0.06 ^d^
C14:1	0.68 ± 0.04 ^a^	0.28 ± 0.05 ^b^	0.08 ± 0.01 ^c^	0.12 ± 0.06 ^c^	0.16 ± 0.01 ^c^	ND
C16:0	38.50 ± 0.87 ^a^	36.71 ± 0.93 ^ab^	33.96 ± 0.7 ^ab^	32.39 ± 7.88 ^ab^	32.68 ± 5.58 ^ab^	30.03 ± 5.03 ^b^
C16:1	ND	ND	2.28 ± 0.17 ^a^	4.11 ± 0.16 ^b^	1.77 ± 0.08 ^c^	3.36 ± 0.02 ^d^
C17:0	1.47 ± 0.01 ^a^	1.01 ± 0.04 ^b^	0.41 ± 0.07 ^c^	0.40 ± 0.04 ^c^	0.42 ± 0.02 ^c^	0.43 ± 0.03 ^c^
C17:1	ND	ND	ND	0.31 ± 0.02 ^a^	0.01 ± 0.01 ^b^	0.23 ± 0.02 ^a^
C18:0	11.76 ± 0.07 ^bc^	12.15 ± 0.76 ^c^	12.03 ± 0.83 ^c^	12.15 ± 1.88 ^c^	9.50 ± 1.41 ^a^	9.86 ± 0.76 ^ab^
C18:1n9t	5.30 ± 0.57 ^a^	0.46 ± 0.11 ^b^	12.81 ± 1.58 ^d^	11.12 ± 1.09 ^cd^	9.27 ± 1.50 ^c^	11.59 ± 2.17 ^cd^
C18:1n9c	20.24 ± 0.18 ^ab^	23.03 ± 0.59 ^a^	14.52 ± 2.54 ^c^	16.88 ± 4.82 ^bc^	17.15 ± 0.51 ^bc^	17.69 ± 3.35 ^bc^
C18:2n6t	ND	ND	0.21 ± 0.13 ^a^	0.43 ± 0.23 ^a^	0.17 ± 0.05 ^a^	0.33 ± 0.03 ^a^
C18:2n6c	1.32 ± 0.06 ^a^	1.08 ± 0.25 ^a^	11.79 ± 2.03 ^b^	11.24 ± 4.56 ^b^	13.58 ± 1.19 ^b^	15.41 ± 2.79 ^b^
C20:0	0.39 ± 0.09 ^b^	0.73 ± 0.11 ^c^	0.19 ± 0.01 ^a^	0.21 ± 0.10 ^ab^	1.09 ± 0.16 ^d^	0.20 ± 0.05 ^a^
C18:3n6	0.11 ± 0.02 ^a^	0.16 ± 0.02 ^a^	0.26 ± 0.11 ^a^	0.32 ± 0.01 ^a^	1.48 ± 0.47 ^b^	0.32 ± 0.05 ^a^
C20:1	0.17 ± 0.02 ^a^	0.11 ± 0.02 ^a^	0.24 ± 0.06 ^a^	0.59 ± 0.11 ^b^	0.50 ± 0.08 ^b^	0.56 ± 0.08 ^b^
C18:3n3	0.30 ± 0.07 ^cd^	0.60 ± 0.02 ^e^	0.19 ± 0.01 ^bc^	0.09 ± 0.06 ^a^	0.08 ± 0.02 ^ab^	0.40 ± 0.03 ^d^
C21:0	ND	0.20 ± 0.11 ^a^	1.38 ± 0.84 ^b^	0.66 ± 0.03 ^ab^	0.76 ± 0.05 ^ab^	0.91 ± 0.15 ^ab^
C20:2	0.74 ± 0.06 ^a^	0.96 ± 0.35 ^ab^	2.58 ± 0.44 ^d^	1.09 ± 0.17 ^abc^	1.33 ± 0.24 ^bc^	1.47 ± 0.09 ^c^
C22:0	0.28 ± 0.01 ^bc^	0.26 ± 0.03 ^b^	0.36 ± 0.05 ^c^	0.52 ± 0.07 ^d^	0.08 ± 0.03 ^a^	0.16 ± 0.02 ^a^
C20:3	ND	ND	0.46 ± 0.06 ^a^	0.53 ± 0.24 ^a^	0.66 ± 0.04 ^ab^	0.83 ± 0.11 ^b^
C22:1	0.20 ± 0.01 ^a^	0.36 ± 0.08 ^ab^	0.56 ± 0.05 ^b^	1.17 ± 0.25 ^c^	1.34 ± 0.01 ^c^	0.51 ± 0.12 ^b^
C24:1n9	0.07 ± 0.01 ^a^	ND	0.02 ± 0.01 ^b^	ND	0.01 ± 0.00 ^c^	0.08 ± 0.05 ^a^
C20:4	0.24 ± 0.17 ^a^	ND	0.82 ± 0.28 ^b^	1.77 ± 0.42 ^c^	1.29 ± 0.14 ^bc^	1.43 ± 0.23 ^c^
C20:5	0.1 ± 0.01 ^a^	0.13 ± 0.01 ^a^	0.82 ± 0.13 ^b^	0.99 ± 0.05 ^c^	0.08 ± 0.02 ^a^	0.15 ± 0.02 ^a^
C24:0	0.23 ± 0.08 ^a^	ND	ND	ND	0.07 ± 0.01 ^b^	ND
C22:6	ND	ND	ND	0.43 ± 0.07 ^a^	0.12 ± 0.01 ^b^	0.35 ± 0.02 ^a^
Sums of fatty acids						
SFA	70.30 ± 0.56 ^a^	72.99 ± 2.3 ^a^	53.18 ± 2.4 ^b^	50.32 ± 10.20 ^b^	51.49 ± 4.8 ^b^	47.57 ± 2.6 ^b^
MUFA	26.61 ± 0.85 ^ab^	24.10 ± 0.27 ^a^	29.71 ± 2.61 ^bc^	32.95 ± 3.94 ^c^	30.2 ± 1.10 ^bc^	32.63 ± 2.44 ^c^
PUFA	2.91 ± 0.17 ^a^	2.90 ± 0.38 ^a^	17.07 ± 2.52 ^b^	16.83 ± 4.43 ^b^	18.19 ± 0.45 ^b^	20.56 ± 3.30 ^b^
n-6	1.69 ± 0.13 ^a^	1.25 ± 0.27 ^a^	13.07 ± 2.19 ^b^	13.59 ± 4.37 ^b^	15.96 ± 0.27 ^b^	17.49 ± 3.11 ^b^
n-3	0.40 ± 0.05 ^a^	0.72 ± 0.01 ^ab^	1.40 ± 0.07 ^bc^	1.70 ± 0.71 ^c^	0.90 ± 0.03 ^ab^	1.71 ± 0.78 ^c^

Values within each row with different superscripts are significantly different (*p* < 0.05). Data are presented as the mean ± standard deviation, *n* = 3. SFA: saturated fatty acid, MUFA: monounsaturated fatty acid, PUFA: polyunsaturated fatty acid, n-6: omega-6 fatty acid (C18:2n-6 + C20:4n-6 + C18:3n-6), n-3: omega-3 fatty acid (C18:3n-3 + C20:5n-3 + C22:6n-3). ND = Not detected.

**Table 4:** The mineral contents of canine, bovine, and caprine milk (mg/100 mL).

	Holstein	Saanen	Golden Retriever	Labrador	Caucasian Sheepdog	Rottweiler
Mg	9.63	14.3	12.8	13	9.85	32.9
K	126.2	152.1	76.9	104.8	113.3	189.4
Na	49.9	60.6	104.5	110.2	80.7	92.1
Fe	ND	ND	ND	0.405	0.58	0.749
Zn	0.31	0.394	1.34	1.13	0.85	0.854
Cu	ND	ND	0.047	0.119	0.046	0.398
Mn (μg/100 mL)	ND	ND	10.6	11.7	16.6	10.6
Se (μg/100 mL)	2.36	1.06	10.6	11	10	39.2

ND = Not detected.

## Data Availability

The datasets generated for this study are available on request to the corresponding author.
